# Profiling Relationships Between Abdominal Pain, Gastrointestinal Symptoms and Pelvic Pain Bothersomeness in Young Females

**DOI:** 10.1111/ajo.70137

**Published:** 2026-04-24

**Authors:** Catherine Von Felten, Caroline Warr, Amy Tinetti, Shannon Murphy, Louise Hug, Oyekoya T. Ayonrinde, Darren Beales, Robert Waller

**Affiliations:** ^1^ School of Allied Health, Faculty of Health Sciences Curtin University Perth Australia; ^2^ Fiona Stanley Hospital Murdoch Australia; ^3^ Medical School The University of Western Australia Perth Australia

**Keywords:** abdominal pain, anxiety, gastrointestinal symptoms, pelvic pain, Raine Study

## Abstract

**Background:**

Abdominal pain, gastrointestinal symptoms and pelvic pain are common complaints of young females seeking healthcare. These symptoms can co‐exist and become recurrent, impacting on quality of life.

**Aim:**

This study investigated the relationships between abdominal pain and gastrointestinal symptoms at 17‐years of age and pelvic pain bothersomeness (PPB) at 22‐years of age in young females.

**Materials and Methods:**

A cross‐sectional observational study utilising 17 and 22‐year Gen2 female data of the Raine Study (*n* = 584). Abdominal pain and gastrointestinal symptoms at 17 years were: frequency, consistency and/or pain of bowel movements, bloating, nausea, vomiting, analgesia for cramps and laxative use in the preceding 3 months. At 22 years, PPB was determined by the Urogenital Distress Inventory Short Form (UDI‐6). Additional health‐related variables were analysed to understand the symptom patterns of participants.

**Results:**

17‐year‐old females, with abdominal pain and gastrointestinal data who answered the UDI‐6 for PPB (*n* = 450); 347 (77%) were not bothered by PPB, 64 (14%) reported mild PPB and 39 (9%) reported moderate–severe PPB at 22‐years (*p* = 0.168). Symptoms of varied stool consistency, vomiting, nausea and laxative use, but not isolated abdominal pain, at 17‐years were significantly associated with PPB at 22‐years. Co‐variants of depression, anxiety, bullying, living with a partner, poor sleep and smoking showed increased prevalence with severity of PPB.

**Conclusion:**

Gastrointestinal symptoms in adolescence were associated with pelvic pain bothersomeness (PPB) in young adulthood. Early detection of abdomino‐pelvic symptoms may be useful to allow early, targeted and multi‐disciplinary management to optimise physical and mental health outcomes.

## 1 Introduction

Abdominal and pelvic pain symptoms were the most reported reasons for emergency department presentation amongst Australian adolescents between 2021 and 2022 [1]. These conditions have high prevalence in young females and can involve symptoms of the urinary, digestive, reproductive, musculoskeletal and nervous systems. The association between pathology and symptomatology in these conditions is often non‐linear, with pain frequently presenting in the absence of discernible pathological findings [2, 3, 4] and subsequent challenges in navigating care options [5, 6].

Abdominal pain is often precipitated by other gastrointestinal symptoms including constipation, diarrhoea, nausea, vomiting and abdominal bloating [7, 8]. Like pelvic pain, a specific causative pathology may not be identified, as demonstrated by Rome IV criteria with the diagnosis of Functional Abdominal Pain Disorders Not Otherwise Specified (FAPD‐NOS) [4, 9]. FAPD‐NOS has a global prevalence of 13.5% for children aged between 4 and 18 years, with increased rates in females (15.9%) compared to males (11.5%) [4]. Recurrent abdominal pain in childhood and adolescence is associated with poor emotional well‐being, feeling anxious or depressed, being bullied at school, poor coping and reduced overall health status [7, 10]. Pelvic pain relates to discomfort in the tissues around the lower abdominal region, pelvis, pelvic organs and genitals [11, 12]. This impacts one in four females of reproductive age worldwide and is associated with poor emotional wellbeing [11, 13]. Self‐reported pelvic pain bothersomeness (PPB) is a recognised measure of the impact and burden of symptoms [3, 14]. In 2023, publicly funded multi‐disciplinary pelvic pain clinics opened around Australia to manage the volume, chronicity and burden of bothersome pelvic pain [15].

There is a need to better understand the relationship between abdominal pain, gastrointestinal (GI) symptoms and PPB and factors associated with their presence [6]. This study aims to profile the relationships between abdominal pain and/or gastrointestinal symptoms at 17 years and PPB at 22 years. Profiling variables related to health, medication use, demographics, sleep and smoking were examined.

## 2 Materials and Methods

### 2.1 Population

This cross‐sectional observational study evaluated data of Generation 2 (Gen2) female participants from the Raine Study. This cohort was reported to be representative of the wider general Western Australian population; details of the original study are described elsewhere [16].

Our sub‐study included females who responded ‘yes or no’ to having abdominal pain at 17 years (*n* = 450) and a valid response to the Urogenital Distress Inventory short form (UDI‐6) at 22 years (*n* = 584). Pain and female pelvic health have been identified as a priority by The Raine Study Community Advisory Committee [3, 5] and the wider population.

### 2.2 Data Collection

The process for data collection at the Gen2‐17 and ‐22 year follow‐ups is outlined elsewhere [2]. The Raine Study data set is covered by a consolidated ethics committee approval from the University of Western Australia Human Research Ethics Committee (RA/4/20/5722). This specific project was approved by the Raine Study (Project licence number #CAR0950.) with specific approval from Curtin University Human Research Ethics Committee (HRE2023‐0586, 10 October 2023, extended annually).

### 2.3 Primary Variables

#### 2.3.1 Abdominal and Gastrointestinal Symptoms at 17‐Years

Abdominal pain is the presence of ‘a belly ache or abdominal pain’ unrelated to menstrual periods in the preceding three months. The participants who answered ‘yes’ to having abdominal pain were questioned further regarding frequency of pain, with options of ‘less than once per month’, ‘1–3 times per month’, ‘once per week’, ‘several times per week or everyday’. Other gastrointestinal symptoms were the frequency of bowel movements, stool consistency, bloating, nausea, vomiting, analgesic use or laxative use, which have been independently associated with abdominal pain in this cohort [2].

#### 2.3.2 Pelvic Pain Bothersomeness at 22‐Years

A combined measure of PPB and prevalence was gathered through the UDI‐6 at the Gen2 22‐year follow‐up [3, 14]. The question, ‘Do you experience, and if so, how much are you bothered by, pain or discomfort in the lower abdomen/genital area?’ had responses of ‘not at all’, ‘slightly’, ‘moderately’, or ‘greatly’. Participants were grouped into three PPB categories: ‘not bothered’ (describing those answering ‘not at all’), ‘mild PPB’ (those answering ‘slightly’) and ‘moderate–severe PPB’ (with combined responses ‘moderately’ and ‘greatly’) [3, 17].

### 2.4 Additional Variables

#### 2.4.1 Health‐Related and Medication Use

Health‐related and medication use variables from the Gen2‐17‐year follow‐up were overall health rating, being bullied at school, analgesic and oral contraceptive pill use. Details of medications, healthcare utilisation and other medical conditions were obtained via a questionnaire completed by a parent or primary caregiver. A binary variable for ‘lifetime prevalence’ of diagnosed menstrual problems, diagnosed depression and diagnosed anxiety was derived by combining the affirmative answers from both 17 and 22 years: ‘yes, now’, ‘yes, now and in the past’, ‘yes, in the past’. The 22‐year variables included from the Gen2‐22‐year follow‐up were medication use for cramps or period pain, and symptoms of anxiety and/or depression measured by the Depression Anxiety Stress Scale (DASS‐21) [18].

#### 2.4.2 Demographic, Sleep and Smoking

Demographic factors from the Gen2‐17 and ‐22 year follow up were included based on their independent association with PPB in prior Raine study analyses [3]. The 22‐year variables were: total usual pay after tax ($AU), relationship and co‐habitant status, sleep quality (measured by Pittsburgh Sleep Quality Index (PSQI)), smoking participation, and waist‐hip ratio (WHR).

### 2.5 Power Calculation

The sample size was predetermined by the Gen2 participants with abdominal pain and gastrointestinal symptoms (17 years) and PPB data (22 years). Adequate power was reached to detect a standardised effect size of ≥ 0.27 with a significance of *p* < 0.05 and a power of 0.8, based on estimated group numbers.

### 2.6 Statistical Analysis

Data was checked for normality of distribution and outliers. The PPB groups were compared with abdominal pain, gastrointestinal symptoms, laxative use, health‐related and medication use, demographics, sleep and smoking data at two time periods, 17‐years and 22‐years. PPB groups were compared using the chi‐square test when comparison variables were categorical. When a comparison variable had a category frequency of less than 10 participants, a Fisher's exact test was used. Waist‐hip ratio was the only continuous variable and was analysed using the Kruskal–Wallis test, due to the data being skewed. For differences in proportions, associations were considered substantive when there was a difference of 10% or more between groups, when comparing each variable where the total prevalence across the whole sample was more than 25% for that variable [19]. For WHR, difference in median/interquartile range (IQR) was used to guide interpretation of a meaningful difference (moderate effect size). A significant association was considered to be a 0.5 or larger effect size (moderate effect size) [19]. The outcomes that were statistically significant but did not have a moderate effect size and were not included in the discussion [20]. Statistical analysis was performed using the Stata/BE 18.0 for Windows (Statacorp LP College Station, TX), with the assistance of a biostatistician.

## 3 Results

### 3.1 Abdominal Pain, Gastrointestinal Symptoms and Laxative Use

Five hundred and eighty‐four females answered the UDI‐6 for PPB at 22‐years, 450 females (77.05%) had abdominal pain and gastrointestinal data at 17‐years reported in Table 1. For those with 17‐year old data, ‘no PPB’ represented 77% (*n* = 347), ‘mild PPB’ had 14% (*n* = 64) and ‘moderate to severe PPB’ with 9% (*n* = 39) of the sample by 22‐years. There was no association reported between PPB groups and isolated abdominal pain in the last 3 months (*p* = 0.168). For ‘consistency of bowel movements’ there was a lower prevalence (*p* = 0.024) of a normal stool type in the ‘moderate to severe PPB’ group (74.4%) compared to ‘no PPB’ (89%) but not the ‘mild PPB’ (82.8%). Reporting ‘unexplained vomiting’ differed between PPB groups (*p* = 0.017), with participants denying unexplained vomiting in the ‘no PPB’ group (91.7%), compared to the ‘moderate to severe PPB’ (80.5%) group but not the ‘mild PPB’(82.8%). There was a higher prevalence (*p* < 0.003) of ‘unexplained nausea’ in the ‘moderate to severe PPB’ (41.5%), ‘mild PPB’ (46.5%) groups compared to the ‘no PPB’ group (26.7%). There was less laxative use reported to ease constipation (*p* < 0.001) in the ‘no PPB’ (98%), ‘mild PPB’ (96.9%) groups compared to the ‘moderate to severe PPB’ (84.6%).

**TABLE 1:** The association of abdominal pain, gastrointestinal symptoms and laxative use (17‐years) with pelvic pain bothersomeness (22‐years).

Group	Whole group	Pelvic pain bothersomeness groups (22‐years)			Overall *p*
		Not bothered	Mild	Moderate–severe	
Variable	Number (%) or median (IQR)	Number (%) or median (IQR)	Number (%) or median (IQR)	Number (%) or median (IQR)	
Pelvic pain bothersomeness	584	450/584 (77.1)	87/584 (14.9)	47/584 (8.0)	
Abdominal pain in the last 3 months at 17‐years					0.168^a^
Valid response	450	347 (77.1)	64 (14.2)	39 (8.7)	
Yes	187 (41.6)	136 (39.2)	31 (48.4)	20 (51.3)	
No	263 (58.4)	211 (60.8)	33 (51.6)	19 (48.7)	
Abdominal pain frequency, if abdominal pain = yes					0.386^b^
Less than once a month	76/186 (40.9)	61 (45.2)	9 (29.0)	6 (30.0)	
1–3 times a month	77/186 (41.4)	52 (38.5)	15 (48.4)	10 (50.0)	
Once a week or more	33/186 (17.7)	22 (16.3)	7 (22.6)	4 (20.0)	
Duration of abdominal pain, if abdominal pain = yes					0.326^b^
< 1 h	69/187 (36.9)	53 (39.0)	8 (25.8)	8 (40.0)	
1–4 h	88/187 (47.0)	60 (44.1)	20 (64.5)	8 (40.0)	
Most of day or greater	30/187 (16.0)	23 (16.9)	3 (9.7)	4 (20.0)	
Bowel movement harder or lumpier than usual, if abdominal pain					0.888^b^
Never	122/185 (65.9)	89 (65.9)	19 (63.3)	14 (70.0)	
Sometimes	57/185 (30.8)	42 (31.1)	10 (33.3)	5 (25.0)	
Most of the time	6/185 (3.2)	4 (3.0)	1 (3.3)	1 (5.0)	
Bowel movement softer or more watery than usual, if abdominal pain = yes					0.372^b^
Never	79/186 (30.1)	57 (42.2)	15 (48.4)	7 (35.0)	
Sometimes	66/186 (25.1)	51 (37.8)	10 (32.3)	5 (25.0)	
Most of the time/always	41/186 (15.6)	27 (20.0)	6 (19.4)	8 (40.0)	
Abdominal pain got better after bowel movement, if abdominal pain = yes					0.752^b^
Never	41/186 (22.0)	30 (22.2)	7 (22.6)	4 (20.0)	
Sometimes	87/186 (46.8)	62 (45.9)	17 (54.8)	8 (40.0)	
Most of the time/always	58/186 (31.2)	43 (31.9)	7 (22.6)	8 (40.0)	
Frequency of bowel action in the last 3 months at 17‐years					0.117^b^
< once per week	17/450 (3.8)	10 (2.9)	6 (9.4)	1 (2.6)	
1–2 times per week	54/450 (12.0)	44 (12.7)	5 (7.8)	5 (12.8)	
3–6 times per week	180/450 (40.0)	145 (41.8)	25 (39.1)	10 (25.6)	
1 time per day	159/450 (35.3)	121 (34.8)	21 (32.8)	17 (15.4)	
> 2 times per day	40/450 (8.9)	27 (7.8)	7 (10.9)	6 (15.0)	
Consistency of bowel movement in the last 3 months at 17‐years					**0.024** ^b^
Stool hard/very hard	41/449 (9.1)	25 (7.2)	7 (10.9)	9 (23.1)	
Stool normal (not too hard/not too soft)	390/449 (86.8)	308 (89.0)	53 (82.8)	29 (74.4)	
Stool soft/mushy/watery	18/449 (4.0)	13 (3.8)	4 (6.3)	1 (2.6)	
Stomach felt bloated in the last 3 months at 17‐years					0.216^b^
Never	68/449 (15.1)	61 (17.6)	4 (6.3)	3 (7.7)	
< once per month	142/449 (31.6)	107 (30.9)	22 (34.4)	13 (33.3)	
1–3 times per month	136/449 (30.3)	104 (30.1)	19 (29.7)	13 (33.3)	
More than once per week	103/449 (22.9)	74 (21.4)	19 (29.7)	10 (25.6)	
Vomiting, no cause at 17‐years					**0.017** ^b^
Not true	380/425 (89.4)	299 (91.7)	48 (82.8)	33 (80.5)	
True	45/425 (10.6)	27 (8.3)	10 (17.2)	8 (19.5)	
Nausea, no cause at 17‐years					**< 0.003** ^b^
Not true	294/425 (69.1)	239 (73.3)	31 (53.5)	24 (58.5)	
True	131/425 (30.8)	87 (26.7)	27 (46.5)	17 (41.5)	
Used laxative in the last 3 months to ease constipation at 17‐years					**< 0.001** ^b^
Yes	15/448 (3.3)	7 (2.0)	2 (3.1)	6 (15.4)	
No	433/448 (96.7)	338 (98.0)	62 (96.9)	33 (84.6)	

*Note:* Data reported as mean or number (%). Bolded *p*‐value = substantive finding.

^a^ Chi‐squared.

^b^ Fisher's Exact.

### 3.2 Health‐Related and Medication Use Variables

The lifetime prevalence of being bullied at school was positively associated with PPB (*p* = 0.014), with moderate to severe PPB (48.8%) and mild PPB (54.2%) compared to no PPB (35.9%) reported in Table 2. Those taking medication for cramps or period pain reported less PPB severity, with moderate to severe PPB (63%), mild PPB (79.8%), and no PPB (78.1%) experienced at 22‐years (*p* = 0.057). The PPB severity with medication use for cramps or period pain became stronger with the co‐existence of abdominal pain at 22‐years (*p* = 0.011).

**TABLE 2:** Health‐related and medication use Gen‐17 & 22‐years summary statistics via pelvic pain bothersomeness (*n* = 584).

Group	Whole group	Pelvic pain bothersomeness groups (22‐years)			Overall *p*
		Not bothered	Mild	Moderate–severe	
Variable	Number (%)	Number (%)	Number (%)	Number (%)	
Pelvic pain bothersomeness	584	450/584 (77)	87/584 (15)	47/584 (8)	
Overall health rating at 17‐years and answered abdominal pain (17‐years)					**0.025** ^b^
Excellent	70/451 (15.5)	62 (17.8)	6 (9.4)	2 (5.1)	
Very good	187/451 (41.5)	139 (39.9)	27 (42.2)	21 (53.9)	
Good	136/451 (30.2)	109 (31.3)	18 (28.1)	9 (23.1)	
Fair	52/451 (11.5)	35 (10.1)	10 (15.6)	7 (17.9)	
Poor	6/451 (1.3)	3 (0.9)	3 (4.7)	0 (0.0)	
Bullied at school at 17‐years Yes	171/431 (39.7)	119/331 (35.9)	32/59 (54.2)	20/41 (48.8)	**0.014** ^a^
Analgesic use in the past six months at 17‐years (prescription or non‐prescription)					
Use, yes	301/483 (62.3)	225/373 (60.3)	49/67 (73.1)	27/43 (62.8)	0.137^a^
Abdominal pain, yes	127/183 (69.4)	90/133 (67.7)	24/31 (77.4)	13/19 (68.3)	0.567^a^
Oral contraception use in the past six months at 17‐years					
Use, yes	158/483 (32.7)	121/373 (32.4)	20/67 (29.9)	17/43 (39.5)	0.557^a^
Abdominal pain, yes	71/183 (38.8)	51/133 (38.3)	9/31 (29.0)	11/19 (57.9)	0.124^b^
Regular medication for cramps or period pain at 22‐years					
Use, yes	131/573 (22.9)	346/443 (78.1)	67 (79.8)	29 (63.0)	0.057^a^
Abdominal pain, yes	46/182 (25.3)	28/134 (20.9)	8/29 (27.6)	10/19 (52.6)	**0.011** ^b^
Diagnosed depression (Lifetime prevalence)					
Yes	156/509 (30.7)	99/386 (25.7)	38/78 (48.7)	19/45 (42.2)	**< 0.001** ^a^
Abdominal pain, yes	58/183 (31.7)	39/29.3 (29.3)	13/41.9 (41.9)	6/19 (31.6)	0.373^b^
Diagnosed anxiety (Lifetime prevalence, *n* = 510, missing = 74)					
Yes	144/510 (28.2)	96/388 (24.7)	33/76 (43.4)	15/46 (32.6)	**0.003** ^a^
Abdominal pain, yes	53/183 (29.0)	36 (27.1)	12/31 (38.7)	5/19 (26.3)	0.463^b^
Diagnosed menstrual problem (Lifetime prevalence, *n* = 503, missing = 81)					
Yes	153/503 (30.4)	99/386 (25.7)	29/70 (41.4)	25/47 (53.2)	**< 0.001** ^a^
Abdominal pain, yes	67/185 (36.2)	42/134 (31.3)	14/31 (45.2)	11/20 (55.0)	0.061^b^
Depression symptoms at 22‐years (DASS‐21) (*n* = 581, missing = 3)					**0.002** ^a^
Normal/Mild (0–13)	450/581 (77.5)	361/448 (80.6)	60/86 (69.8)	29/47 (61.7)	
Moderate/Severe (> 13)	131/581 (22.5)	87/448 (19.4)	26/86 (30.2)	18/47 (38.3)	
Anxiety symptoms at 22‐years (DASS‐21) (*n* = 581, missing = 3)					**< 0.001** ^a^
Normal/Mild (0–9)	452/581 (77.8)	365/448 (81.5)	58/86 (67.4)	29/47 (61.7)	
Moderate/Severe (> 9)	129/581 (22.2)	83/448 (18.5)	28/86 (32.6)	18/47 (38.2)	

*Note:* Data reported as mean or number (%), Bolded *p*‐value = substantive finding.

^a^ Chi‐squared.

^b^ Fisher's Exact.

Lifetime prevalence of diagnosed depression was associated with PPB (*p* < 0.001), with moderate to severe PPB (42.2%), mild PPB (48.7%) and no PPB (25.7%). The lifetime prevalence of diagnosed anxiety differed between PPB groups (*p* = 0.003) with moderate to severe PPB (32.6%) and mild PPB (43.4%) compared to no PPB (24.7%) Co‐exist abdominal pain did not strengthen the association of lifetime diagnosed depression or anxiety (*p* = 0.373 and *p* = 0.463 respectively).

The lifetime prevalence of diagnosed menstrual problems was significantly higher across PPB groups (*p* < 0.001), with moderate to severe PPB (53.2%), mild PPB (41.4%), and no PPB (25.7%) experienced at 22‐years. Abdominal pain co‐existing with diagnosed menstrual problems was not statistically significant (*p* = 0.061).

### 3.3 Demographic, Sleep and Smoking Data

Relationship status and living situation at 22 years varied between groups with a higher prevalence of PPB severity when a participant was in a relationship and living with a partner (*p* < 0.001) reported in Table 3. For those in a relationship and living together, 52.2% reported moderate–severe PPB, compared to 33.3% in the mild PPB and 23.7% in the no PPB groups. In contrast, for those who reported being single, 21.8% reported moderate–severe PPB, 26.4% reported mild PPB and 42.4% reported no PPB. Sleep quality significantly varied between PPB groups (*p* < 0.001), 61.4% with moderate–severe PPB, 46.3% with mild PPB and 30.7% with no PPB (30.7%) reported poor sleep quality respectively. 86.6% of the cohort answered no to smoking with significant differences between PPB groups (*p* = 0.004). There were less non‐smokers in the moderate–severe PPB group (72.3%) compared to the mild PPB (82.8%) and no PPB group (88.8%).

**TABLE 3:** Demographic, sleep and smoking data summary statistics with pelvic pain bothersomeness.

Group	Whole group	Pelvic pain bothersomeness groups (22‐years)			Overall *p*
		Not bothered	Mild	Moderate–severe	
Variable	Number (%) or mean (SD)	Number (%) or mean (SD)	Number (%) or mean (SD)	Number (%) or mean (SD)	
Pelvic pain bothersomeness	584	450/584 (77)	87/584 (15)	47/584 (8)	
Total usual pay per week after tax at 22‐years (*n* = 544, missing = 40)					0.531^a^
≤ $604	319/544 (58.6)	249/418 (59.6)	46/79 (58.2)	24/47 (51.1)	
> $605	225/544 (41.4)	169/418 (40.4)	33/79 (41.2)	23/47 (48.9)	
Relationship status (*n* = 581, missing = 3)					**< 0.001** ^a^
Single	223/581 (38.4)	190/448 (42.4)	23/87 (26.4)	10/46 (21.8)	
In a relationship but not living together	199/581 (34.3)	152/448 (33.9)	35/87 (40.2)	12/46 (26.1)	
In a relationship and living together	159/581 (27.4)	106/448 (23.7)	29/87 (33.3)	24/46 (52.2)	
Sleep quality at 22‐years (*n* = 541, missing = 43)					**< 0.001** ^a^
Good	350/541 (64.7)	289 (69.6)	44 (53.7)	17 (38.6)	
Poor	191/541 (35.3)	126 (30.7)	38 (46.3)	27/44 (61.4)	
Smoking (yes) (*n* = 581, missing = 3)					**0.004** ^a^
Yes	78/581 (13.4)	50 (11.2)	15 (17.2)	13 (27.7)	
No	503/581 (86.6)	397 (88.8)	72 (82.8)	34 (72.3)	
Waist‐hip ratio at 22‐years (*n* = 477, missing = 107)	0.80 (0.1)	0.80 (0.07)	0.80 (0.07)	0.79 (0.07)	0.828^b^

*Note:* Bolded *p*‐value substantive finding.

^a^ Chi‐squared.

^b^ Linear regression.

## 4 Discussion

This study investigated the association between abdominal pain, gastrointestinal symptoms and PPB in young females living in Australia. Gastrointestinal symptoms of varied stool consistency, unexplained nausea, vomiting and laxative use to treat constipation, but not isolated abdominal pain, at 17 years were independently associated with increased PPB. This study demonstrates that five years after experiencing gastrointestinal symptoms, young females frequently experience PPB. When abdominal pain was experienced at 17 years and females were diagnosed with a menstrual problem or taking regular medication for cramps/period pain at 22 years, it was associated with increased prevalence and severity of PPB at 22 years.

Higher PPB in young females is significantly associated with a lifetime prevalence of anxiety and/or depression in young females. These results support previous Raine Study research that PPB is associated with increased levels of anxiety and depression [3] and this relationship has also been demonstrated in children and adolescents with abdominal pain [2]. This suggests that a cluster of physical or psychological symptoms that coexist with abdominal symptoms as well as PPB. Additional health‐related or demographic variables associated with increased PPB severity included smoking, poor sleep quality, and being in a co‐habitant relationship or married at 22 years. The increased PPB severity and association with co‐habitant/marriage relationship may have a confounding variable such as frequency of sexual intercourse which was not analysed. Persistent pain disorders are associated with changes to the peripheral and central nervous systems through altered hypothalamic pituitary–adrenal (HPA) axis regulation, cortisol production and reduced immune response [21]. This may be further compounded by nicotine withdrawal symptoms in smokers [22], poor sleep quality [21], fatigue, reduced mental health and significant negative impacts on engagement in education, work, relationship and social domains [23, 24].

### 4.1 Strengths and Limitations

A strength of this study is the large population‐based cohort, lowering selection bias and being representative of the general population. This provides insight into the complex nature of abdominal and gastrointestinal symptoms which may contribute to the sequelae of abdominal pain and PPB [8, 25].

Limitations included only having one validated question from the UDI‐6 to represent PPB. The comparison of the cohorts at two different time points limited the interpretation of this study due to no available data of PPB at 17‐years and no abdominal pain data at 22‐years. Capture of key variables at multiple time points would have allowed for prospective representation of more complex relationships between abdominal pain, gastrointestinal symptoms and PPB.

### 4.2 Interpretation in Relation to the Literature

Abdominal pain, gastrointestinal symptoms and PPB are common reasons for health seeking in young females and often coexist with reduced physical and emotional well‐being [1, 2, 3, 5]. This research demonstrates specific clinical features which may be associated with persistent pain include partaking in smoking, depression, anxiety, poor health status, poor sleep, hard stool type, laxative use and unexplained nausea and vomiting. The clustering of abdomino‐pelvic pain, gastrointestinal symptoms and reduced emotional wellbeing identifies individuals at risk of reduced long‐term physical and mental health outcomes.

Functional disorders associated with abdominal pain impact approximately 1 in 4 people in the United States and are often associated with chronic pain syndromes, such as migraine, depression, fibromyalgia, pelvic pain, reduced quality of life and increased health care utilisation [26, 27]. A recent meta‐analysis and systematic review analysed 14 studies investigating abdominal pain in the primary care setting, with a mean prevalence of 2.8% for abdominal pain in GP consultations [25]. The cause of the abdominal pain was not identified in approximately one third of cases. This study outlines the clinical difficulty of having minimal diagnostic guidelines for abdominal pain and a high rate of cases remaining unexplained or cases spontaneously resolved.

A systematic review investigating the co‐morbidity of irritable bowel syndrome (IBS) with other disorders found a median of 49.9% of women were found to have co‐morbid IBS with chronic pelvic pain [28]. A large prospective cohort study of young female adolescents in 2021 found that endometriosis was associated with a 5‐fold increase in odds of irritable bowel syndrome, with acyclic pain being a strong predictor of IBS [27]. Almost half of the pelvic pain or endometriosis sufferers reported via an online survey that they had delayed or postponed assessments and almost a quarter had withdrawn [29]. Pelvic pain (and PPB) may profoundly impact one's quality of life through reduced attendance at school, work, social engagements and subsequent reduced opportunities for future pursuits.

Pelvic pain can influence multiple body systems: reproductive, neurological, musculoskeletal, urological, gastrointestinal, endocrine, vascular [3, 5]. Bi‐directional cross‐organ sensitisation and viscero‐somatic convergence have been described as a potential mechanism [23, 30].

### 4.3 Clinical Relevance and Future Research

Young adulthood is a critical time of development, the early detection and profiling of abdominal, gastrointestinal and pelvic pain symptoms is important for clinicians to consider when assessing women and will encourage targeted individualised care and result in better outcomes [16, 24]. This cluster of symptoms may start early in life and common underlying mechanisms may be an important consideration for patient‐centred care [2, 25]. There is growing interest in the field of pain and female pelvic health, with pelvic pain being named as one of the top five health concerns in Australia [24]. The Australian Government has allocated $58.1 million to the National Women's Health strategy 2020–2030 and the establishment of multi‐disciplinary pelvic pain clinics around the country [15].

After the exclusion of red flags and specific conditions, the screening of abdomino‐pelvic and GI symptoms could enable early profiling of pain sub‐types. This could enhance patient understanding, agency and reduce distress for those experiencing symptoms. It may also allow targeted symptom management, support and self‐efficacy to reduce long‐term health burden [3, 4] More longitudinal studies to explore the underlying mechanisms behind pain manifestations and altered nociceptive processing are required to better understand the contributing factors leading to persistent pain. These conditions require a holistic and multidisciplinary approach, as recommended by clinical practice guidelines and systematic reviews for better quality of care [4].

## Funding

The core management of the Raine Study is funded by The University of Western Australia, Curtin University, The Kids Research Institute Australia, Women and Infant Research Foundation, Edith Cowan University, Murdoch University, The University of Notre Dame Australia and the Western Australian Future Health Research and Innovation Fund (Grant ID WACSOSP2023‐2024, WACSOSP2025/7). The Raine Study Gen2‐17 year follow‐up was funded by NHMRC Program Grants 353514 and 403981 and Abdominal Ultrasound data from the Gastroenterological Society of Australia and Fremantle Hospital, Perth. The Raine study Gen2‐22 year follow‐up was funded by NHMRC project grants 1027449, 1044840 and 1021858. Funding was also generously provided by Safe Work Australia. There was no additional funding obtained for this specific research project.

## Ethics Statement

The Raine Study data set is covered by a consolidated ethics committee approval from the University of Western Australia Human Research Ethics Committee (RA/4/20/5722). This specific project was approved by the Raine Study (Project Licence #CAR0950). Institutional ethics committee approval was obtained from the Princess Margaret Hospital for Children Human Research Ethics Committee. This specific project has obtained Human Research Ethics Committee approval by Curtin University Research Ethics Committees (HRE2023‐0586 on 10 October 2023 and annually renewed thereafter).

## Consent

Signed informed consent from The Raine Study participants at 22 years old, and parents or legal guardians and adolescent consent at 17 years were obtained for the original study. The data collected was coded to remain anonymous, posing no threat to confidentiality (2).

## Conflicts of Interest

The authors declare no conflicts of interest. L.H. was a participant involved in the Raine Study at the time that data was collected but was not involved in any decisions made by the Raine Study during the project approval process.

## Data Availability

The individual‐level data from the Raine Study cannot be made available or stored on another, non‐Raine Study public repository due to legal and ethical constraints. The Raine Study is a publicly available data provider and data can be accessed by interested researchers upon reasonable request. The licence for this project is #CAR0950.
